# Protective effect of hydroxysafflor yellow A on dopaminergic neurons against 6-hydroxydopamine, activating anti-apoptotic and anti-neuroinflammatory pathways

**DOI:** 10.1080/13880209.2020.1784237

**Published:** 2020-07-13

**Authors:** Xiaomei Yang, Yun Li, Lin Chen, Mingguo Xu, Jianbo Wu, Peng Zhang, Deon Nel, Baozhu Sun

**Affiliations:** a Department of Anesthesiology, Qilu Hospital, Cheeloo College of Medicine, Jinan, P.R. China; b Department of Traditional Chinese Medicine, Dezhou People’s Hospital, Dezhou, P.R. China; c Department of Pharmacology, School of Medicine, Cheeloo College of Medicine, Shandong University, Jinan, P.R. China; d Department of Pediatric Cardiology, Shenzhen Children’s Hospital, Shenzhen, P.R. China

**Keywords:** Parkinson’s disease, apoptosis, p38 MAPK pathway

## Abstract

**Context:**

Hydroxysafflor yellow A (HSYA) has been shown to have neuroprotective effects in cerebral infarction. However, its underlying roles in apoptosis and inflammation in Parkinson’s disease (PD) are unknown.

**Objective:**

The present study investigates the effects and underlying mechanisms of HSYA on dopaminergic (DA) neurodegeneration, inflammation, and apoptosis.

**Materials and methods:**

The PD model was established by 2 μL of 6-hyroxydopamine (6-OHDA) (3 μg/μL) striatal injection in C57BL/6J mice with different doses of HSYA (2, 4, or 8 mg/kg). *In vitro,* after being treated with HSYA for 1 h, SH-SY5Y cells were exposed to 6-OHDA for 24 h before analysis. Expression of tyrosine hydroxylase (TH) in substantia nigra (SN) and corpus striatum (STR) was evaluated by immunohistochemistry (IHC) and western blot. In addition, apoptosis-related and inflammatory proteins were examined by western blot.

**Results:**

Administration of HSYA significantly reduced the Apomorphine (APO)-induced rotation, decreased from 122.5 ± 15.1 (6-OHDA group) to 47.2 ± 14.3 (8 mg/kg HSYA group). HSYA partially restored a deficit in the SN and STR of PD mice brains in TH. Furthermore, western blot analysis revealed that HSYA reduced inflammatory proteins, including iNOS, COX-2 and NF-κB and attenuated the elevation of DA neuronal apoptosis observed in PD*. In vitro* assays showed that HSYA reduced the levels of p-p38 and p-JNK and increased that of p-ERK in 6-OHDA-leisoned SH-SY5Y cells.

**Conclusions:**

These findings indicate that HSYA protects against 6-OHDA induced DA neurodegeneration partly by regulating the MAPK inflammatory signalling pathway and apoptosis which highlight its therapeutic potential in the treatment of PD.

## Introduction

Parkinson’s disease (PD) is a debilitating age-related motor neurodegenerative disease that imposes a substantial social burden worldwide and significantly impacts patients’ quality of life. The motor symptoms characteristic of this disease, such as bradykinesia, tremor, and rigidity (Mullin and Schapira 2015; Kabra et al. 2018), have been linked to the degeneration of nigrostriatal dopaminergic neurons. In recent years, increased attention has been given to the roles played by inflammation and apoptosis in the development of PD pathophysiology (Ahmed et al. 2012; Chao et al. 2014; Chattopadhyaya et al. 2015). Accumulating evidence suggests that neuroinflammation and oxidative stress can contribute to the progression of PD (Loeffler et al. 2017; Gelders et al. 2018). Similarly, apoptosis may play an important role in PD pathogenesis (Winslow and Rubinsztein 2011; Ahmed et al. 2012). As a result, immunomodulatory strategies targeting these processes have become attractive options for therapies seeking to attenuate PD progression (Hirsch and Hunot 2009). However, the molecular mechanisms at play are still poorly understood.

**Figure 1.**
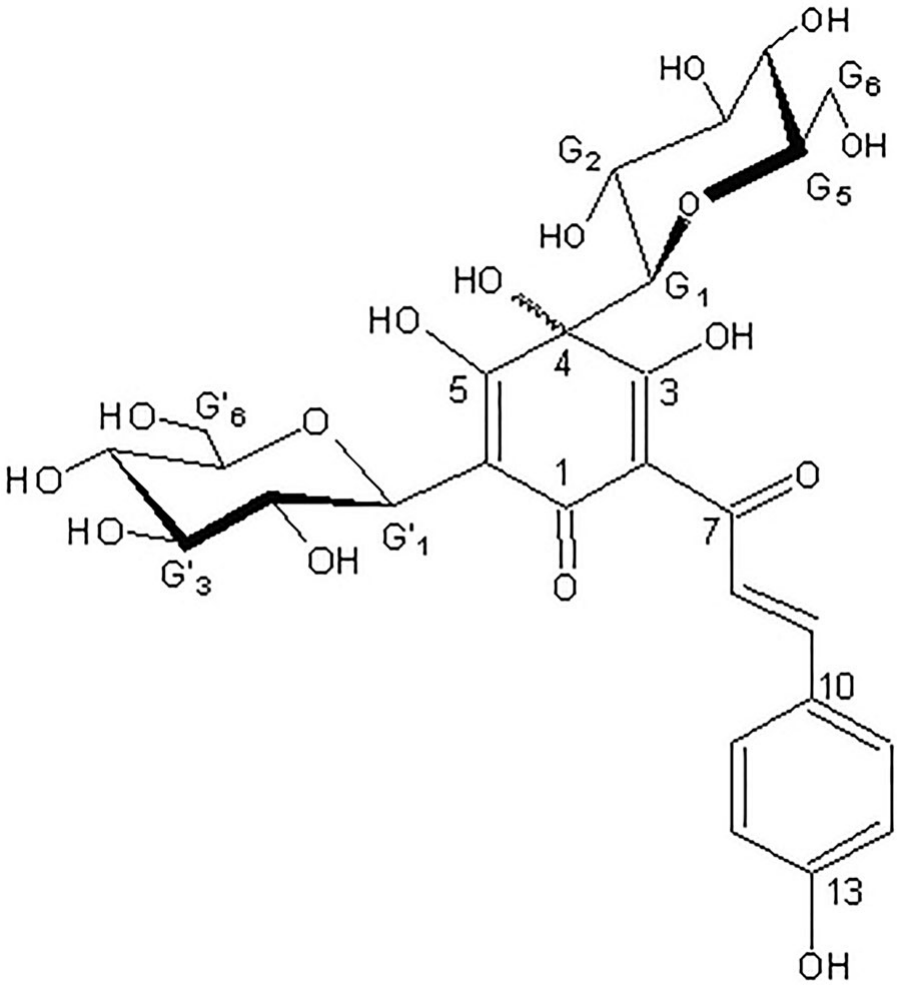
Chemical structure of HSYA.

*Carthamus tinctorius* L. (Compositae) also named safflower which is a medicinal plant was officially listed in the Chinese Pharmacopoeia (named in Honghua). It has been used in the treatment of cerebral and cardiovascular disease in Chinese folk medicine for hundreds of years (Guan et al. 2013). Hydroxysafflor yellow A (HSYA) is a natural pigment extracted from *Carthamus tinctorius* (Figure 1), and it has become a major focus of recent research due to its protective effects in neurodegenerative, cardiovascular, and cerebrovascular diseases (Wei et al. 2005; Han et al. 2009; Sun et al. 2012; Pei et al. 2017; Wang et al. 2017). Previous studies have shown that the neuroprotective effect of HSYA is associated with the attenuation of oxidative stress, the inhibition of apoptosis (Chen et al. 2013), and the alleviation of inflammation (Pei et al. 2017). Moreover, HSYA has been demonstrated to protect motor function and dopamine neuron integrity in a rodent model of PD (Han et al. 2013). We previously reported that HSYA exhibits protective effects towards cerebral ischemia-reperfusion (I/R) injury by suppressing thrombin production (Sun et al. 2010) and through an antioxidant activity (Wei et al. 2005). However, the molecular mechanisms associated with its protective effects against neuronal cell inflammation and apoptosis in PD remain poorly understood.

In this study, we characterise the protective role of HSYA in PD. We investigated HSYA’s effect on the degeneration of PD-associated dopaminergic neuronal cells and characterised its implication at the molecular level in neuronal cell inflammation and apoptosis through the MAPK and cytochrome C/caspase-3 signalling pathways, respectively.

## Materials and methods

### Isolation and identification of compounds

Hydroxysafflor yellow A (Figure 1), with a molecular formula of C_27_H_32_O_16_ (purity 98%), is a yellow amorphous powder extracted from *Carthamus tinctorius*. All chemicals and reagents used in this study were of analytical grade and obtained from Sigma (St. Louis, MO, USA) unless otherwise mentioned.

### Animals

Male C57BL/6J mice (6–8 week, 18–22 g) were purchased from the Experimental Animals Centre of Shandong University as described previously (Chen et al. 2013) and placed in a climate-controlled room maintained on a 12 h light-dark cycle. The experiments were performed according to the Shandong University Guide for the Care and Use of Laboratory Animals.

### Experimental design

All mice were randomly assigned into five groups (15 per group) including a sham group, a 6-hydroxydopamine (6-OHDA) group, and three groups with different doses of HSYA (2, 4, or 8 mg/kg). The HSYA dosage was determined based on the results of our previous study (Chen et al. 2013). Mice were administered HSYA (2, 4, or 8 mg/kg) or saline intraperitoneally fifteen minutes before 6-OHDA injection and subsequently once a day for 14 days.

### 6-OHDA striatal injections

A well-established *in vivo* mouse PD model was used as described previously (Han et al. 2013). After adaption for seven days, the mice were anaesthetised with a 50 mg/kg sodium pentobarbital intraperitoneal (IP) injection and placed in a stereotaxic device. Then, 2 μL of 6-OHDA (3 μg/μL, Sigma-Aldrich, H4381) dissolved in sterile normal saline (NS) with 0.02% ascorbic acid or NS alone were injected into the following sites of the right striatum (STR): point A (2.1 mm lateral, 1.0 mm anterior to the bregma, and 2.9 mm from the dura mater) and point B (2.3 mm lateral, 0.3 mm posterior to the bregma and 2.9 mm from the dura mater), as described previously (Chattopadhyaya et al. 2015). The rate of injection was 0.5 μL/min and following injection the needle was maintained in place for 4 min before being slowly removed. We slowly injected NS alone into the STR of mice in the control group.

### Apomorphine (APO)-induced rotation test and tyrosine hydroxylase (TH) immunohistochemistry

Fourteen days after 6-OHDA injection, apomorphine hydrochloride (0.1 mg/kg) was subcutaneously injected into mice. Before rotations were recorded, the mice were placed in a plastic beaker and adapted to the environment for 5 min. The total number of full 360° rotations in the contralateral direction was counted for 30 min, and the results were reported as average rotations per 10 min over the 30 min measurement period.

For immunohistochemistry detection, the mice were anaesthetized with sodium pentobarbital 14 days after 6-OHDA administration, after which their brains were perfused with 0.9% NS and fixed with 4% paraformaldehyde. Subsequently, the brains (SN and STR) were dissected and fixed in 4% paraformaldehyde for 24 h. Frozen brain tissues were then sectioned into 20 μm thick slices and mounted onto slides after being cryopreserved in 30% sucrose for 48 h. The sections were then incubated with an anti-TH primary antibody (diluted 1:200), then treated with a biotinylated secondary antibody at 37 °C for 1 h, which was followed by an incubation in streptavidin ABC solution. Bright field microscopy (Olympus) was performed to visualise the numbers of positive cells at a 400× magnifications after diaminobenzidine staining (Vector Laboratories).

### Cell culture and CCK8 assay

Human neuroblastoma (Lundblad et al. 2005) SH-SY5Y cells from the American Type Culture Collection (Rockville, MD, USA）were grown in 96-well plates DMEM F12 medium with 10% heat-inactivated foetal bovine serum and 100 U/mL penicillin/streptomycin at 37 °C under an atmosphere with 5% CO_2_. Stock solutions of 6-OHDA were freshly prepared before each experiment in L-ascorbic acid (0.2%). After being treated with HSYA (1, 5, or 10 × 10^−6 ^mol/L) for 1 h, cells were exposed to 50 × 10^−6 ^mol/L 6-OHDA for 24 h before analysis. To measure cell viability, the culture medium was replaced and CCK-8 solution was added to the wells of the 96-well plates, which were then incubated at 37 °C for 1.5 h according to the manufacturer’s instructions. Subsequently, the absorbance was determined at 450 nm using a microplate reader (Tecan, Maennedorf, Switzerland) and was reported as the percentage of viable cells compared to the normal group.

### Western blot assay

Total proteins were extracted from right SN and STR of the mouse brain areas and 6-OHDA-induced SH-SY5Y cells using RIPA extraction reagent (Beyotime, Haimen, China) as previously described (Chen et al. 2013), and the protein concentrations were determined using a Bicinchoninic Acid Protein Assay kit (Thermo Fisher Scientific, Inc., USA). Subsequently, 50 μg of protein samples were separated by sodium dodecyl sulphate polyacrylamide gel electrophoresis (SDS-PAGE) (12%) and transferred onto PVDF membranes (Bio-Rad, Hercules, CA, USA). The PVDF membrane was then incubated at 4 °C overnight with primary antibodies against the following primary proteins: tyrosine hydroxylase (TH) (diluted 1:2000; MAB318, Millipore), pro-caspase-3 (diluted 1:1000; BS9865, Bioworld), COX-2 (diluted 1:500; 12375, Proteintech), Bax (diluted 1:2000; 50599, Proteintech), Bcl-2 (diluted 1:1000; 12789, Proteintech), iNOS (diluted 1:500; 18985, Proteintech), cytochrome C (diluted 1:1000; 10993, Proteintech), cleaved caspase-3 (diluted 1:500; BS7004, Bioworld), NF-κB p-p65 (diluted 1:500; 3033, Cell Signalling Technology), NF-κB p65 (diluted 1:1000; 10745 Proteintech), IκBα (diluted 1:1000; BS3601, Bioworld), p-ERK (diluted 1:1000; 4370, Cell Signalling Technology), ERK **(**diluted 1:2000; 4695, Cell Signalling Technology), p-JNK (diluted 1:1000; 9255, Cell Signalling Technology), JNK (diluted 1:2000, 9252; Cell Signalling Technology), p-p38 (diluted 1:1000; 9216, Cell Signalling Technology), p38 (diluted 1:2000; 9212, Cell Signalling Technology), or GAPDH (diluted 1:2000; 10494, Proteintech). Membranes were then incubated with appropriate HRP-conjugated secondary antibodies at room temperature for 1 h. Subsequently, the membrane was visualised with ECL chemiluminescence reagent (ECL, Millipore) and imaged using a BioRad Chemi-Doc gel system.

### Statistical analysis

All results are presented as the means ± S.E.M. and were analysed using SPSS (version 20.0 for Windows). The statistical analysis of differences among groups was analysed by One-way ANOVA followed by Duncan’s multiple range test. A value of differences was considered significant at *p* < 0.05.

## Results

### HSYA protects against dopaminergic neurodegeneration in PD mice

**Figure 2.**
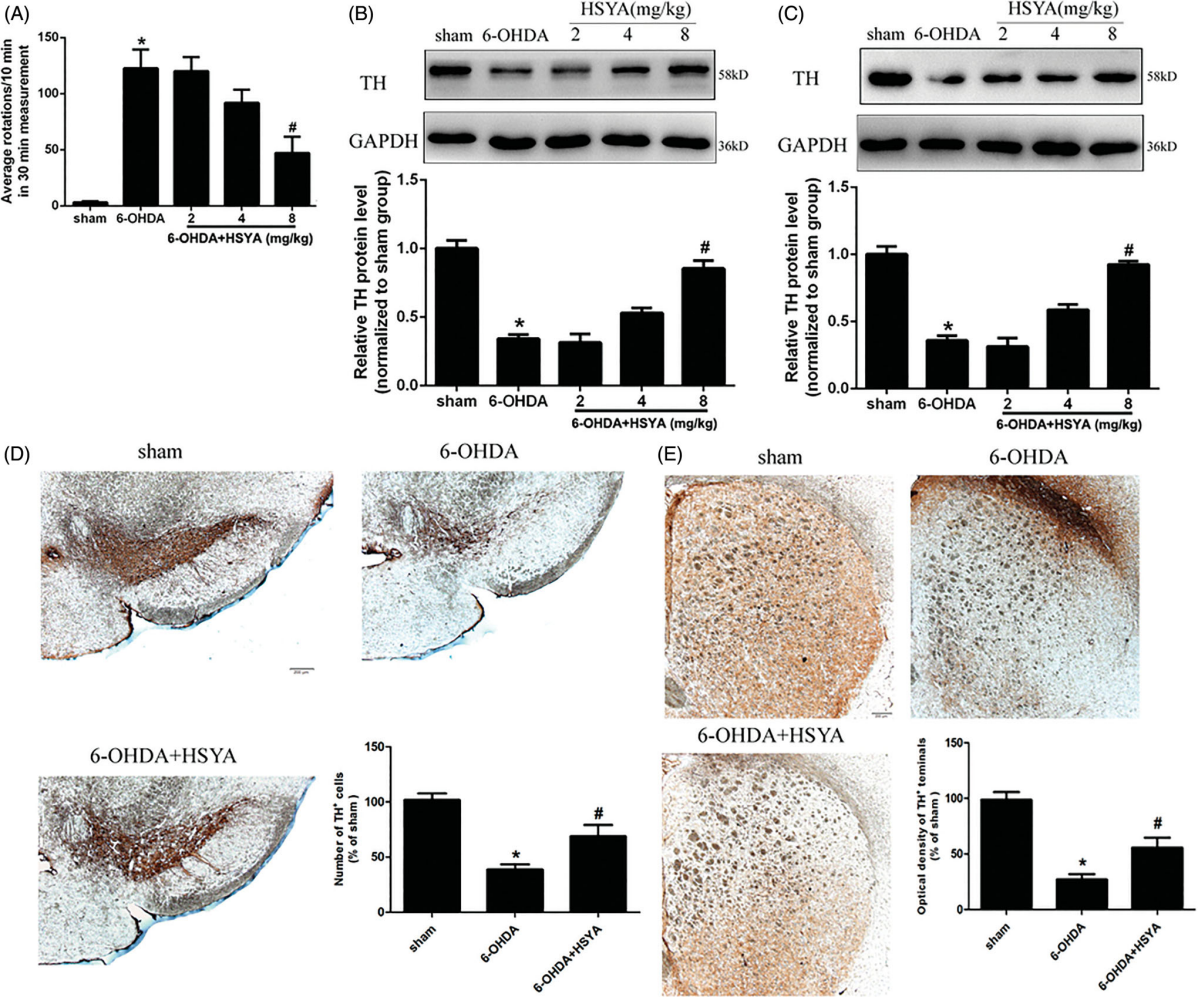
Neuroprotective effects of HSYA in PD mice model. Mice were injected with 6-OHDA into the right striatum in the absence or presence of HSYA at 2, 4, and 8 mg/kg, respectively. (A) APO-induced rotation was measured as the average circle number per 10 min for a period of 30 min following subcutaneous APO injection (*n* = 15 in each group). (B and C) Western blotting was performed to evaluate the expression of TH protein in the SN (B) and STR (C) (*n* = 5 in each group). (D and E) Brain tissue sections were immunohistochemically stained for TH in both the SN (D) and STR (E). The graph bars show the number of TH-positive neurons in the SN (D) and the optical density of TH positive terminals in the STR (E). The data are presented as the means ± S.E.M (*n* = 5 in each group). *p < 0.05 vs. sham group; ^#^*p* < 0.05 vs. 6-OHDA group.

To determine whether HSYA protects against dopaminergic dysfunction associated with PD, we first conducted APO-induced rotation tests (Wei et al. 2005). Contralateral rotation is related to the deterioration of motor performance and the degree of dopaminergic neuron dysfunction (Zheng et al. 2019). As shown in Figure 2(A), APO-induced contralateral rotation was increased in the 6-OHDA group (122.5 ± 15.1) compared with the sham group (3.0 ± 0.9) (*p* < 0.01), indicative of dopaminergic neurodegeneration. Remarkably, the 6-OHDA-induced increase in APO-induced contralateral rotation was significantly attenuated in the HSYA (8 mg/kg) group (47.2 ± 14.3) compared with 6-OHDA group (122.5 ± 15.1) (*p* < 0.01).

To further characterise the effect of HSYA on dopaminergic neuron deficit, we analysed the expression of TH, a key neuron-specific enzyme required for dopamine synthesis. Western blot analysis showed that TH protein levels were reduced in both the SN and STR in the 6-OHDA group compared to those observed in the sham group. Notably, HSYA treatment attenuated the 6-OHDA-induced reduction of TH in both the SN and STR (Figure 2(B,C)). Accordingly, the *in situ* immunohistochemistry staining results for TH showed a significant decrease in the number of TH-positive neurons in the SN (38.7 ± 4.7 *vs.* 101.7 ± 6.0) and decreased the optical density of TH-positive terminals (27.0 ± 4.9 *vs.* 98.7 ± 7.0) of the 6-OHDA group compared to that observed in the sham group, and HSYA treatment (8 mg/kg) attenuated the decrease observed in the SN (68.7 ± 10.4 *vs.* 38.7 ± 4.7) (Figure 2(D)) and increased the optical density of TH-positive terminals in the STR (55.7 ± 9.0 *vs.* 27.0 ± 4.9) (Figure 2(E), *p* < 0.05). Taken together, these results indicate that 6-OHDA striatal injection induces a significant depletion of TH and that HSYA treatment reverses this phenomenon.

### HSYA attenuates dopaminergic neuron apoptosis in the SN of PD mice

**Figure 3.**
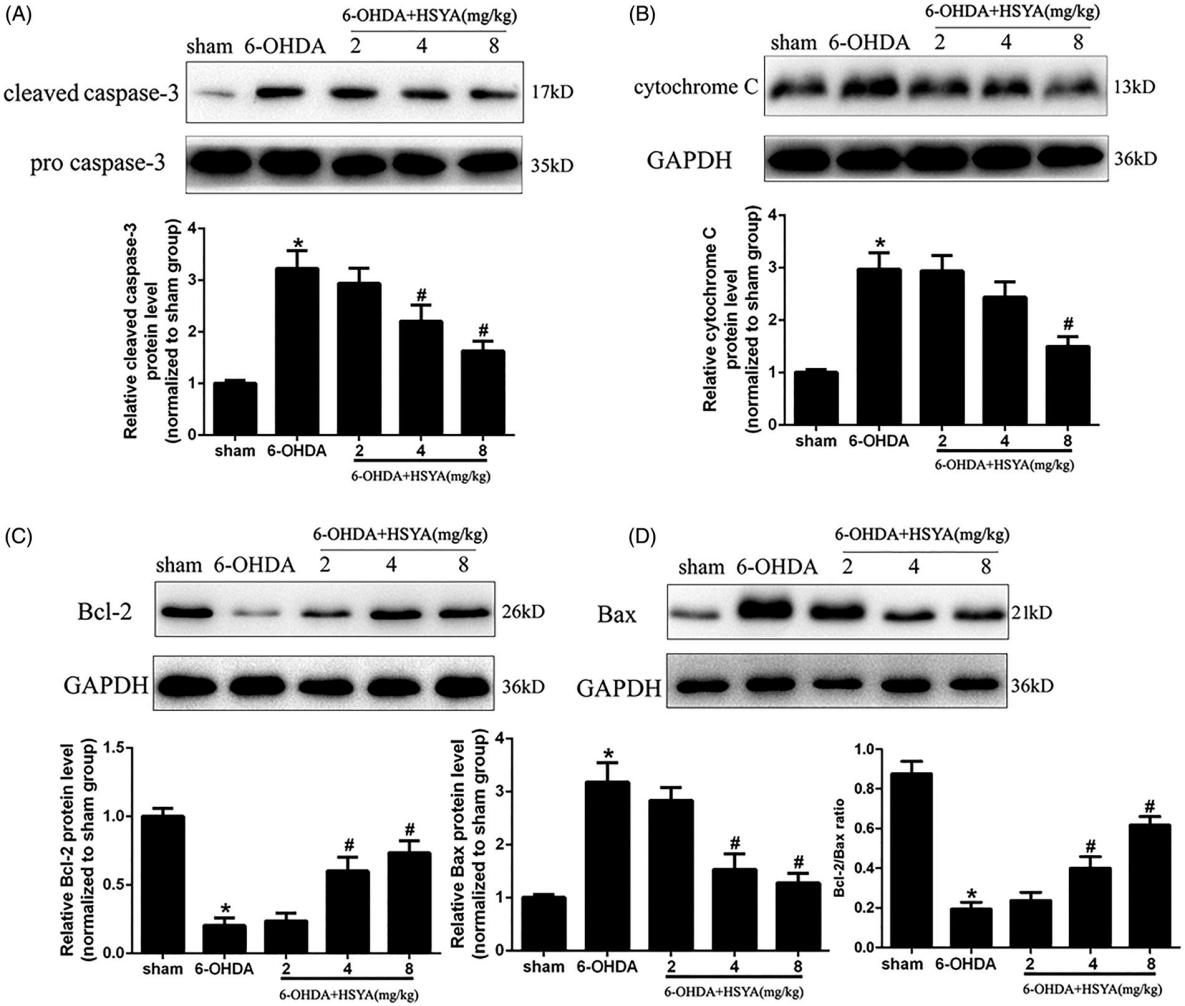
HSYA administration alleviates apoptosis in PD mice model. Western blotting was performed to evaluate the effect of HSYA on the levels of caspase-3 (A), cytochrome C (B), Bcl-2 (C) and Bax (D). The data are presented as the means ± S.E.M. (*n* = 5 in each group). *p < 0.05 vs. sham group; ^#^*p* < 0.05 vs. 6-OHDA group.

To examine the role of HSYA in apoptosis, we assessed the expression levels of Bcl-2, Bax, caspase-3, and cytochrome C in the SN by western blot analysis. The results presented in Figure 3(A,B) show that the caspase-3 and cytochrome C protein levels were significantly increased in the 6-OHDA group compared with those observed in sham group and that this increase was inhibited by HSYA treatment in a dose-dependent manner. Furthermore, as shown in Figure 3(C,D), the expression of Bcl-2 was significantly decreased while that of Bax was increased significantly in the 6-OHDA group compared with that observed in the sham group. These changes in Bcl-2 and Bax protein levels were significantly reduced and the Bcl-2: Bax protein ratio increased significantly with HSYA treatment at dosages of 4 and 8 mg/kg, respectively (*p* < 0.05).

### HSYA attenuates neuroinflammation in the SN of PD mice

**Figure 4.**
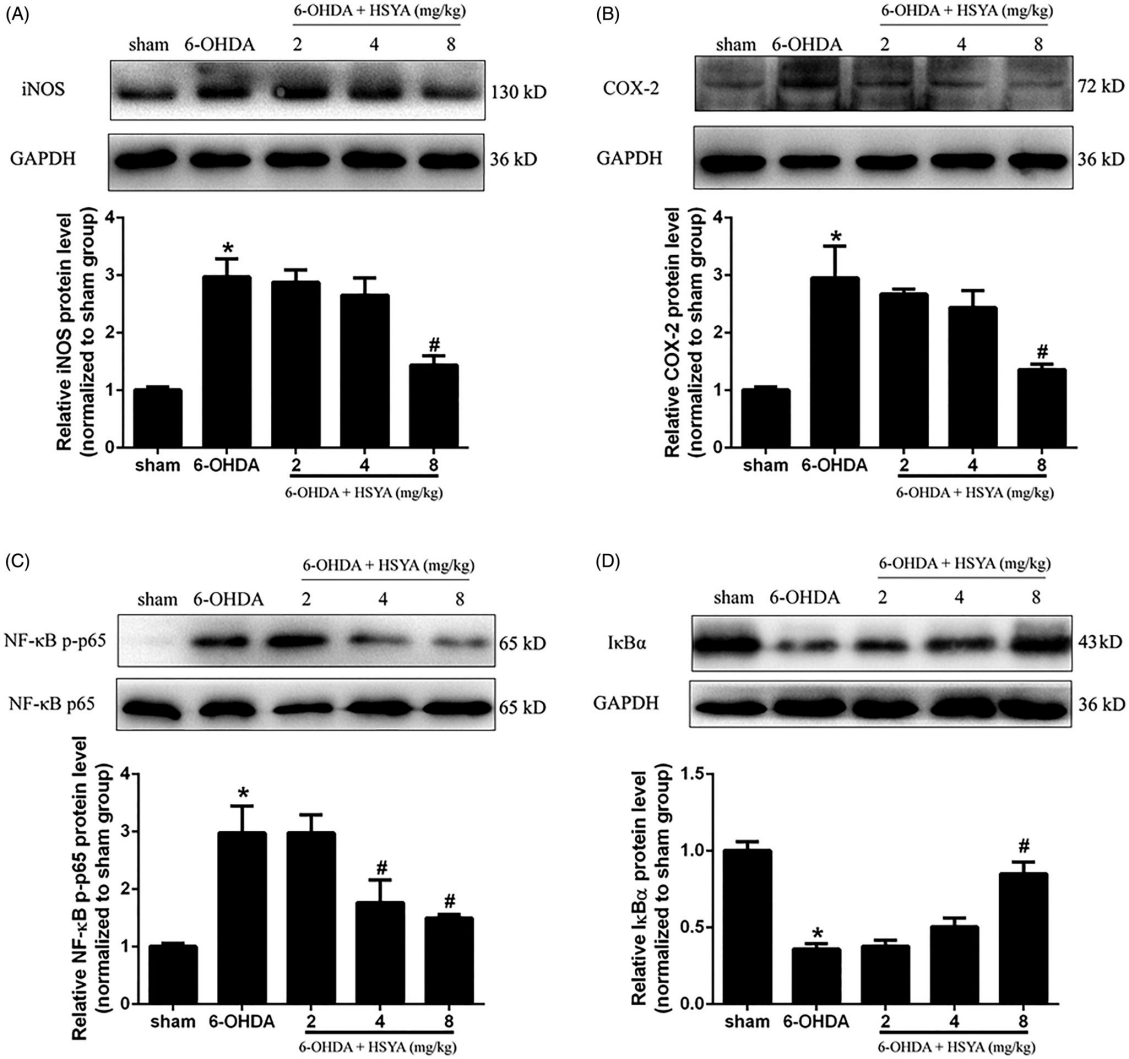
HSYA administration inhibits the activation of COX-2, iNOS and NF-κB in PD mice model. C57BL/6 mice were intraperitoneally injected with 2 μL of 6-OHDA (3 μg/μL) into the right STR in the presence or absence of HSYA. After injection, western blotting was performed to evaluate the effect of HSYA on the activation of iNOS (A) and COX-2 (B) in the sham, 6-OHDA, and HSYA groups. The levels of NF-κB p-p65, NF-κB p65(C), and IκBα (D) triggered by 6-OHDA and HSYA treatments in the PD model were measured by western blotting. The data are presented as the means ± S.E.M *p < 0.05 vs. sham group; ^#^*p* < 0.05 vs. 6-OHDA group.

To evaluate the protective effects of HSYA on neuroinflammation, we determined the levels of COX-2, iNOS, and NF-κB/IκBα proteins in the SN by western blot analysis. As shown in Figure 4, the 6-OHDA group had elevated levels of iNOS, COX-2, and NF-κB and reduced levels of IκBα compared with the sham group. However, HSYA (8 mg/kg) significantly decreased the levels of iNOS, COX-2 and NF-κB proteins and increased those of IκBα, compared to the 6-OHDA group (*p* < 0.05). These findings strongly suggest that HSYA may exert its neuroprotective effects via the modulation of neuroinflammation.

### HSYA reduces inflammation in the SN by mediating MAPK pathways

**Figure 5.**
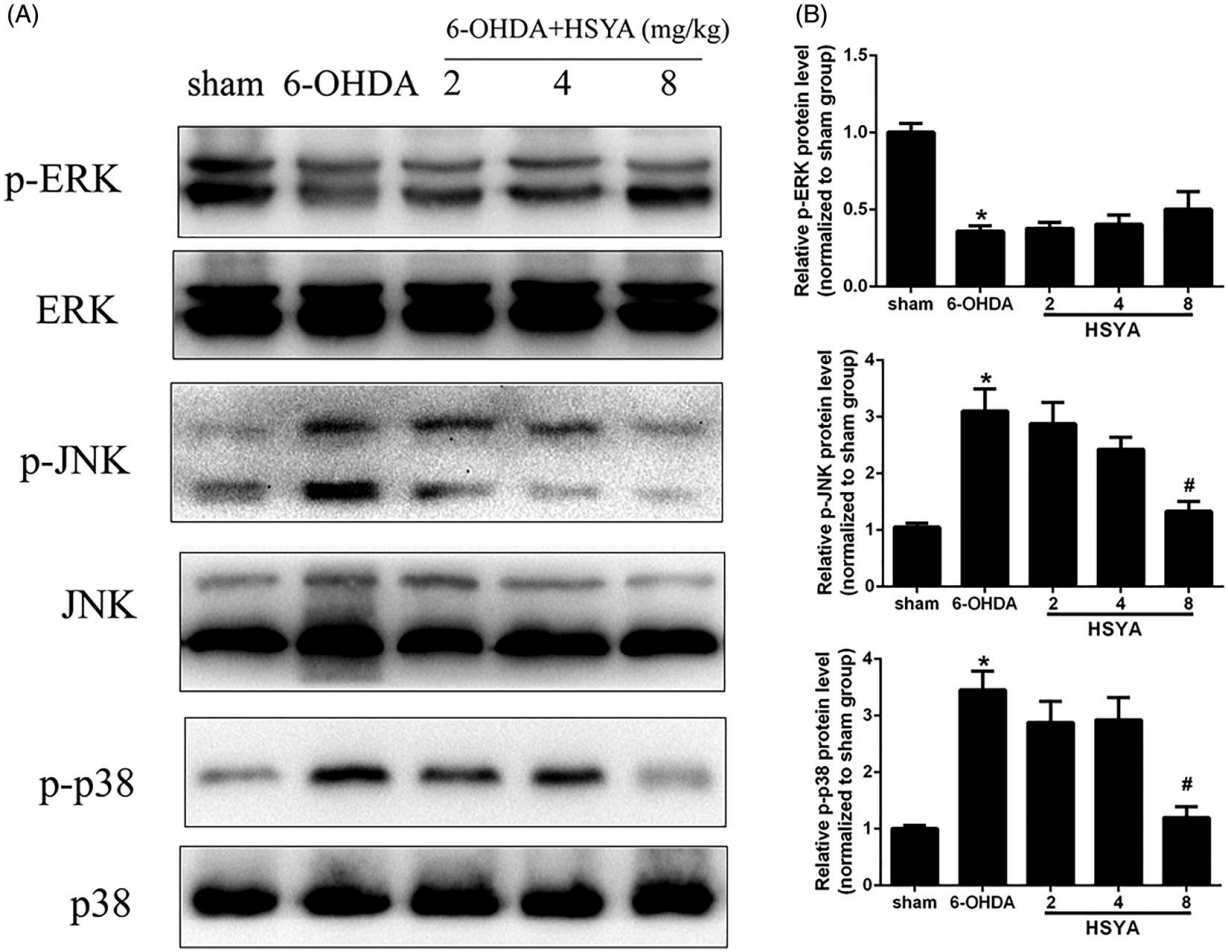
Effect of HSYA on the levels of p-ERK, p-JNK, and p-p38 in PD mice model. C57BL/6 mice were intraperitoneally injected with 2 μL of 6-OHDA (3 μg/μL) into the right STR in the presence or absence of HSYA. After injection, western blotting analysis was performed to evaluate the levels of ERK, p-ERK, JNK, p-JNK, p38 and p-p38 in the SN (A). The graph bars show the levels of p-JNK, p-p38 and p-ERK normalized to those observed in the sham group (B). The data are presented as the means ± S.E.M (*n* = 5 in each group). *p < 0.05 vs. sham group; ^#^*p* < 0.05 vs. 6-OHDA group.

Accumulating evidence has shown that MAPKs are crucial mediators of inflammation, eliciting the activation of the NF-κB pathway and the release and synthesis of proinflammatory mediators (Zheng et al. 2019). To determine whether the modulation of neuroinflammation exerted by HSYA may involve kinases in this pathway, we examined the levels of p-ERK, p-KNK, and p-p38 in the SN by western blot analysis. As shown in Figure 5(A,B), the 6-OHDA group had significantly reduced levels of p-ERK and substantially enhanced levels of p-JNK and p-p38 compared with the sham group. Notably, the levels of p-JNK and p-p38 were reduced (*p* < 0.05) in the HSYA group (8 mg/kg) while the level of p-ERK was elevated in the HSYA (8 mg/kg) group with no statistical significance. Collectively, our results suggest that the anti-neuroinflammatory effect of HSYA is partly mediated through MAPK signalling pathway.

### HSYA attenuates 6-OHDA-induced phosphorylation of p38 and JNK in SH-SY5Y cells

**Figure 6.**
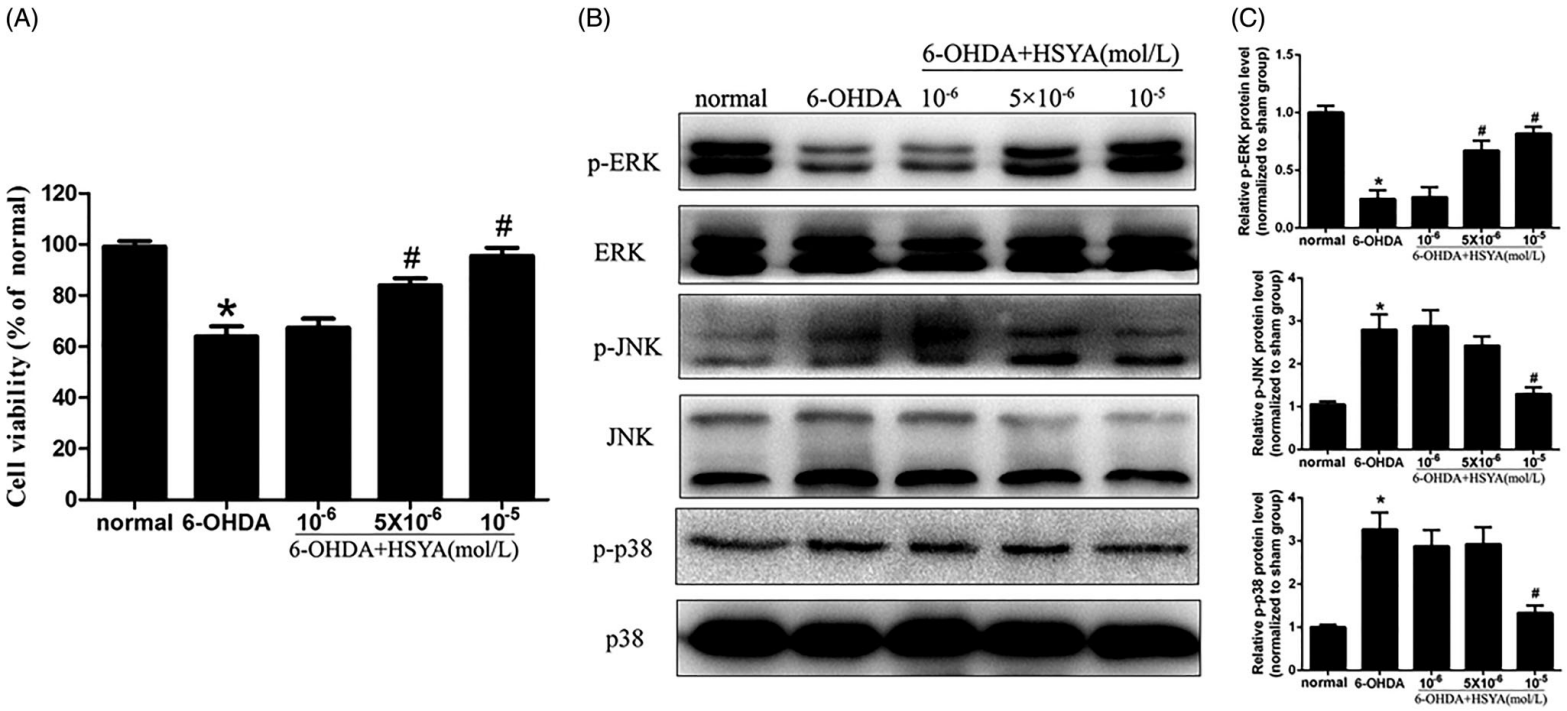
Effect of HSYA on the levels of p-JNK, p-ERK, and p-p38 in 6-OHDA treated SH-SY5Y cells. Cells were treated with HSYA (1, 5, or 10 × 10^−6^ mol/L) for 1 h and exposed to 50 × 10^−6^ mol/L 6-OHDA for 24 h before analysis. A CCK-8 assay kit was used to measure cell viability (A). ERK, p-ERK, p38, p-p38, JNK and p-JNK levels were evaluated by western blotting. The levels of p-ERK, p-JNK and p-p38 were shown as the amount of p-JNK, p-ERK and p-p38 expression normalized to those of JNK, ERK, and p38, respectively (B and C). The data are presented as the means ± S.E.M (*n* = 5 in each group). *p < 0.05 vs. Normal group. ^#^*p* < 0.05 vs. 6-OHDA group.

**Figure 7.**
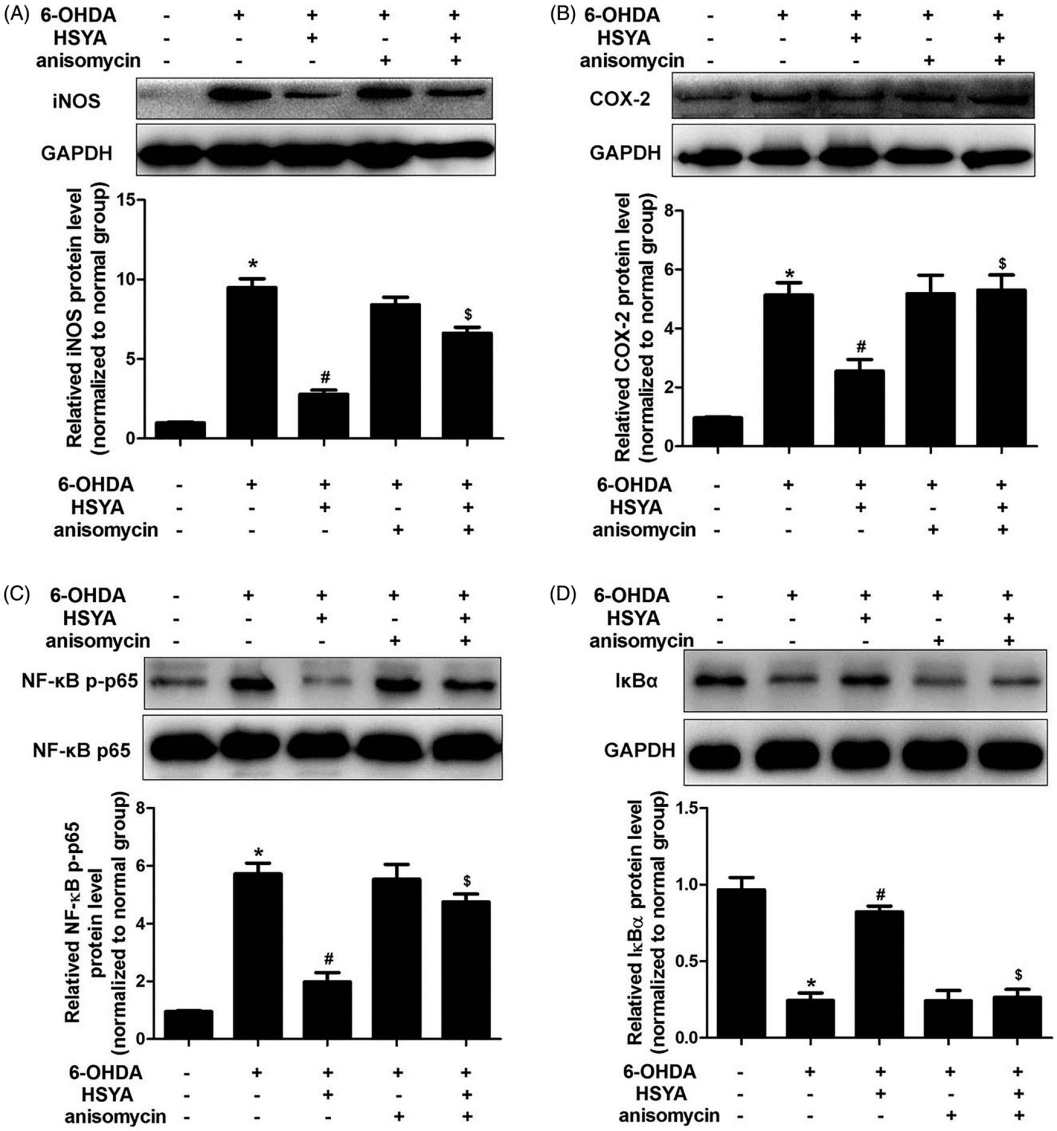
The HSYA-mediated attenuation of 6-OHDA-induced damage is associated with the phosphorylation of JNK in SH-SY5Y cells. Cells were treated with NS or HSYA (10^−5^ mol/L) for 1 h and exposed to 50 × 10^−6^ mol/L 6-OHDA in the absence or presence of anisomycin for an additional 24 h before analysis. iNOS (A), COX-2 (B), NF-κB p-p65 (C), and IκBα (D) levels were evaluated by western blotting. The graph bars show the amount of iNOS (A), COX-2(B), NF-κB p-p65 (C), and IκBα (D) proteins normalized to those observed in the normal group. The data are presented as the means ± S.E.M (*n* = 5 in each group). *p < 0.05 vs. Normal group. ^#^*p* < 0.05 vs. 6-OHDA group. ^$^*p* < 0.05 vs. 6-OHDA + HSYA group.

The CCK8 assay was used to assess the viability of SH-SY5Y cells pre-incubated or not with HSYA prior to treatment with 6-OHDA. As shown in Figure 6(A), in the absence of HSYA preincubation, cell viability was significantly reduced in the 6-OHDA group compared to the normal group. This effect was significantly reversed when cells were preincubated with HSYA prior to 6-OHDA treatment (5 and 10 × 10^−6 ^mol/L, respectively; *p* < 0.05). Furthermore, compared with the 6-OHDA group, HSYA (10 × 10^−6 ^mol/L) treatment notably elevated p-ERK protein level and alleviated those of p-p38 and p-JNK in SH-SY5Y cell (Figure 6(B,C)). These data suggest that HSYA may provide neuroprotective effects in SH-SY5Y cells by regulating MAPK signalling pathway. As shown in Figure 7, HSYA (10 × 10^−6 ^mol/L) significantly decreased the levels of iNOS, COX-2 and NF-κB and increased that of IκBα in SH-SY5Y cells, compared with the 6-OHDA group. Furthermore, the JNK agonist anisomycin was observed to potentially reverse the HSYA-mediated alleviation of inflammation response triggered by 6-OHDA, indicating that the neuroprotective effect of HSYA may be dependent on the phosphorylation of JNK.

## Discussion

The results of the present study showed that HSYA significantly reduced the levels of iNOS, COX-2, and NF-κB and inhibited neuronal apoptosis based on measurements of cytochrome C, caspase-3, Bax, and Bcl-2 in PD mice. We also observed that HSYA regulated MAPK pathway, as indicated by observed reductions in the levels of p-p38 and p-JNK proteins both *in vivo* and *in vitro*. These findings suggest that HSYA protects against 6-OHDA-induced injury in DA neurons by reducing apoptosis and inflammation via the MAPK signalling pathway.

In traditional Chinese medicine, HSYA has been proved to have neuroprotective activity during acute cerebral infarction and antioxidative effects both *in vivo* and *in vitro* (Han et al. 2013; Tonges et al. 2018). Recent studies have reported that HSYA may attenuate both LPS-induced lipopolysaccharide (LPS)-induced human alveolar epithelial cells (Han et al. 2013) and ischemia-reperfusion brain injury in a rat model by modulating inflammatory pathways (Tonges et al. 2018). In addition, the results of another study indicated that HSYA may protect against hypoxic injuries by regulating the apoptosis pathway in endothelial cells of the human brain (Sun et al. 2012). However, the exact underlying mechanisms of the neuroprotective effect of HSYA in PD remains poorly elucidated. The results of our present study showed that HSYA treatment (8 mg/kg) significantly increased the number of TH-positive neurons in the SN and the optical density of TH-positive terminals in the STR. Moreover, the levels of TH in both the SN and the STR were significantly increased with HSYA treatment (8 mg/kg). Additionally, HSYA administration improved APO-induced rotation in a dose-dependent manner. Taken together, the above results indicate that HSYA exerts protective effects against 6-OHDA-induced injury in the dopaminergic neurons of PD.

Apoptosis has been closely implicated in PD and several studies indicate that it may contribute to tissue damage in pathologies and DA neuronal loss in PD (Song et al. 2013; Yang et al. 2018). Caspase-3 is a cysteine protease that plays a key role in apoptosis, and the activation of caspase-3 is associated with a series of signal transduction cascades, including those involving Bax, Bcl-2, and cytochrome C proteins. Bcl-2 is an anti-apoptotic factor that is important for cell survival, whereas Bax promotes apoptosis, and the regulation of apoptosis involves a balance between these proteins (Michaelidis et al. 1996; Zhang et al. 2007; Leverson et al. 2015), which form Bax/Bcl-2 heterodimers, as well as caspase-3 activation. The results of our study showed that HSYA treatment at doses of 4 and 8 mg/kg significantly decreased the levels of caspase-3, cytochrome C, and Bax and increased the level of Bal-2 compared with that observed in the 6-OHDA group. Moreover, the ratio of Bcl-2 and Bax protein was significantly increased after HSYA treatment. Taken together, these results suggest that HSYA may protect dopaminergic neurons against 6-OHDA by reducing apoptosis.

Neuroinflammation is also involved in the initiation and progression and plays an essential role in neuronal degeneration associated with PD (Chao et al. 2014). Inflammatory mediators (iNOS and COX-2) and other proinflammatory cytokines (IL-6 and TNF-α), play crucial roles in the initiation and amplification of inflammatory responses in PD. In addition, NF-κB, which induces the release of inflammatory cytokines in PD, has a potential role in neuronal damage. Furthermore, evidence has also indicated that NF-κB activity is involved in mediating microglia-associated DA degeneration (Kim et al. 2015). Inhibiting the overproduction of these proinflammatory cytokines and mediators was shown to decrease PD-related neuronal damage (Wei et al. 2005; Hirsch and Hunot 2009). Interestingly, previous studies have indicated that HSYA can suppress the expression of TLR-4, TNF-α, IL-6, and IL-1βand to decrease the nuclear translocation of NF-κB p65 in human alveolar epithelial cells (Lv et al. 2016). Our results also showed that the activation of NF-κB, as evidenced by assessing p65 phosphorylation and IκBα degradation, was significantly inhibited in the HSYA group compared with that observed in the 6-OHDA group both *in vivo* and *in vitro*. In addition, the levels of inflammatory cytokines including iNOS and COX-2 were decreased in the HSYA treatment group. These findings indicate that HSYA may exert its neuroprotective effects by alleviating inflammation in PD.

The results of previous studies also suggest that the MAPK signalling pathway participates in the progression of PD (Wei et al. 2005; Zhang et al. 2007). Consistent with these findings, the results of our present study show that 6-OHDA injection significantly enhances p-JNK and p-p38 levels compared with those observed in the sham group and these effects were reversed by HSYA treatment (8 mg/kg). HSYA pre-treated SH-SY5Y cells were subsequently injured with 6-OHDA to further investigate the involvement of MAPK pathway in the protective effects exerted by HSYA against neuronal injury. The results showed that pre-incubation with HSYA enhanced the level of p-ERK and decreased the levels of p-JNK and p-p38 in SH-SY5Y cells as compared with those observed in mice treated with 6-OHDA alone. Moreover, our in vitro data showed that the JNK agonist anisomycin reversed the alleviation of the inflammation response triggered by 6-OHDA in the presence of HSYA indicating that the neuroprotective effect of HSYA may be partly dependent on JNK phosphorylation.

## Conclusions

The results of our study demonstrate that HSYA protects against 6-OHDA-induced injury in DA neurons by reducing the levels of apoptosis and inflammation and that this effect may be partly due to the activation of MAPK signalling pathways. These results provide insights into the molecular mechanisms involved in HSYA protective effects and highlight its therapeutic potential in the treatment of PD.
